# Templated Growth of Crystalline Mesoporous Materials: From Soft/Hard Templates to Colloidal Templates

**DOI:** 10.3389/fchem.2019.00022

**Published:** 2019-01-30

**Authors:** Lei Zhang, Lei Jin, Ben Liu, Jie He

**Affiliations:** ^1^Jiangsu Key Laboratory of New Power Batteries, Collaborative Innovation Center of Biomedical Functional Materials, School of Chemistry and Materials Science, Nanjing Normal University, Nanjing, China; ^2^Department of Chemistry, University of Connecticut, Mansfield, CT, United States; ^3^Institute of Materials Science, University of Connecticut, Mansfield, CT, United States

**Keywords:** mesoporous materials, templates, transition metal oxides, nanoconfinement, polymer micelles, organosilicate

## Abstract

Mesoporous non-siliceous materials, in particular mesoporous transition metal oxides (*m*-TMOs), are of interest due to their fascinating electronic, redox, and magnetic properties for a wide range of applications in catalysis and energy storage. Control of the porosity (e.g., pore size, wall thickness, and surface area) and the crystalline degree (e.g., phase composition, crystallinity, and crystal grain size) of *m*-TMOs are critical for those applications. To crystallize TMOs, high temperature annealing is often needed to remove the amorphous defects and/or tune the compositions of different crystalline phases. This has brought many challenges to surfactant or block copolymer templates used in the process of evaporation-induced-self-assembly to prepare *m*-TMOs. In this review, we summarize the most recent achievements including the findings in our own laboratory on the use of organosilicate-containing colloids for the templated growth of mesoporous materials. We review a few key examples of preparing crystalline mesoporous oxides using different templating methods. The colloidal templating method by which mesoporous nanostructures can be stabilized up to 1,000°C is highlighted. The applications of *m*-TMOs and meso metal-oxide hybrids synthesized using organosilicate-containing colloidal templates in photocatalysis and high-temperature catalysis are also discussed.

## Introduction

Mesoporous materials are porous materials with periodically ordered pores in the range of 2~50 nm. They have large accessible surface areas and tunable pore sizes, which are of particular benefit for mass transport and dispersion of electrons/reactants. Therefore, mesoporous materials show great potential in electrocatalysis (Wu et al., 2012; Ren et al., 2013; Xin et al., 2018), photocatalysis (Li et al., 2007; Onozuka et al., 2012; Joo et al., 2013; Liu et al., 2015b) and energy storage, and conversion (Li et al., 2015, 2016; Fang et al., 2016; Le et al., 2017). Since ordered mesoporous silica such as MCM-41 were discovered in 1992 (Kresge et al., 1992), extensive efforts have been devoted to the development of many other mesoporous silica such as SBA-15/16 (Zhao et al., 1998a,b; Sakamoto et al., 2000), KIT-6 (Kleitz et al., 2003), AMS (Qiu and Che, 2011; Han and Che, 2013), and FDU-12 (Fan et al., 2003, 2005). These mesoporous silica with ordered and interconnected nanopores as well as excellent thermal stability have been broadly used as support for metal nanocatalysts. Other than mesoporous silica, there has been a substantial amount of interest in mesoporous transition metal oxides (*m*-TMOs) due to their unique *d*-shell electrons, resulting in a redox active surface on top of the nanosized pores (Schüth, 2001). Compared to amorphous mesoporous silica, *m*-TMOs exhibit enhanced electronic and optical properties (Kondo and Domen, 2008), which have been proven to be essential in the applications such as photocatalysis (Kuo et al., 2015; Liu et al., 2015b), electrocatalysis (Burke et al., 2015; Song et al., 2018), and battery materials (Su et al., 2016; Zheng et al., 2018).

The crystallinity of *m*-TMOs is of critical importance for some of their applications. Since high-temperature annealing is the most common way to increase the crystallinity and tune the crystalline phases of *m*-TMOs (Lee et al., 2008; Zhang et al., 2010; Liu et al., 2015b), how to stabilize porous frameworks under high temperature is the key to producing highly crystalline *m*-TMOs. Using photocatalysis as an example, TiO_2_ is one of the most well-studied photocatalysts. The low-crystalline TiO_2_ with amorphous domains is known to effectively trap photo-excited electrons and holes that lower the overall utilization of photoexcitation (Ohtani et al., 1997). TiO_2_ has two common crystalline structures, including rutile and anatase. As a photocatalyst, anatase shows much better activity compared to rutile. Interestingly, TiO_2_ with mixed phases outperforms both pure phases (Cong and Xu, 2011; Zhao et al., 2013; Siah et al., 2016). P25 (Degussa) consisting of ~80% of anatase and ~20% of rutile has often been used as a benchmark TiO_2_ photocatalyst. Since there is a type II band alignment of ~0.4 eV (the energy of valance band) (Scanlon et al., 2013), TiO_2_ with a mixture of phases shows enhanced charge separation efficiency at the interface of rutile and anatase (Kawahara et al., 2002; Miyagi et al., 2004). Converting anatase or non-crystalline to rutile TiO_2_ requires high-temperature annealing, e.g., at 800°C, although the rutile TiO_2_ is more stable. When the annealing is carried out in a mesoporous TiO_2_, the collapse of the porous framework occurs before the phase transition due to the overgrowth of crystal grains (Schüth, 2001; Yun et al., 2001). Another example is the formation of crystalline mixed oxides in order to control the optical properties. Carter et al. have proposed the MnO:ZnO alloys that potentially shift the band energy of MnO dramatically (Kanan and Carter, 2012, 2013). MnO as an n-type semiconductor has a large bandgap of 4.0 eV but, when forming alloys with ZnO, its band energy can be shifted largely to a bandgap of 2.6 eV, which is potentially useful for photocatalytic water splitting or CO_2_ reduction. The formation of mixed oxides of MnO:ZnO needs high-temperature treatment at 700°C (Song et al., 2015). The synthesis of such binary or ternary oxides (e.g., copper tungstate and perovskites) in mesoporous form will be very difficult because of the poor thermal stability of porous frameworks. Overall, the control of crystallinity and crystal phases is critical in tuning the physicochemical properties of *m*-TMOs.

**Figure 1:**
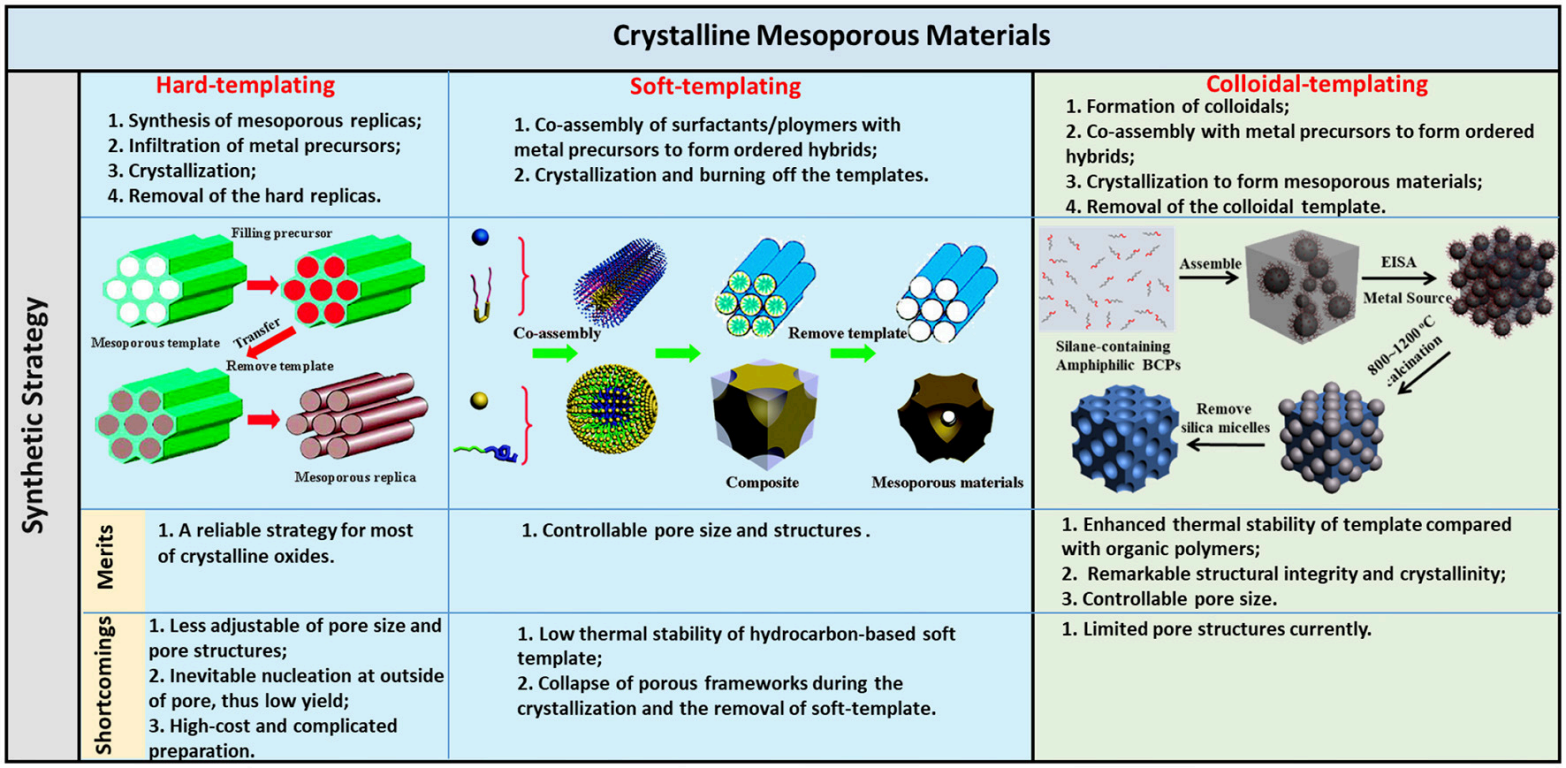
Illustration of the synthetic methods/procedures of crystalline mesoporous materials and the merits/shortcomings of the three methods. Reprinted with permission from Gu and Schuth (2014) and Liu et al. (2015b). Copyright 2015 American Chemical Society and Copyright 2014 Royal Society of Chemistry.

In this review, we summarize the synthetic methods in recent years for the preparation of highly crystalline *m*-TMOs. The synthesis will be discussed by classifying the synthetic methods based on the templates (Figure 1), including hard templates, soft templates, and colloidal templates. There are a number of excellent review papers on the synthesis of mesoporous materials such as TiO_2_ (Li et al., 2013), non-siliceous oxides (Gu and Schuth, 2014), and metal oxides (Ren et al., 2012) by mainly soft-templating and hard-templating methods. We intend to highlight a few key examples in control of the crystallinity of *m*-TMOs by different methods, with a focus on the findings in our own laboratory on the use of organosilicate-containing colloids for the templated growth of mesoporous materials. The applications of colloidal-templated porous oxides and metal-oxide hybrids in photocatalysis will be included. At the end of this review, we include a short perspective in this area. We hope that this review will help readers to understand the challenges and solutions in the rational design and application of crystalline *m*-TMOs.

## Hard-Templating Method

The hard-templating method, also known as nanocasting, provides the most accessible strategy to synthesize crystalline mesoporous materials. Nanocasting is to make use of a rigid mold with defined porous structures on the nanometer scale where the target materials or their precursors are added to fill the pore of the mode, and the primary mold is subsequently removed after the formation of target materials (Lu and Schüth, 2006). In detail, nanocasting of *m*-TMO includes three steps: (1) synthesis of mesoporous replicas (e.g., silica, carbons, and aluminates); (2) infiltration of metal precursors and further decomposition to form crystalline materials; and (3) removal of the hard template to release the pores. Upon removal of the mold, the yielded *m*-TMO will replicate the complementary mesostructures of the hard template. For instance, silica templates equipped with ordered cylindrical channels can produce nanowires or nanoarrays (Lu and Schüth, 2005), while the template with spherical pores can prepare the nanosphere arrays (Lu and Schüth, 2006), and the template with bicontinuous pores results in bicontinuous mesostructured duplicates with periodically and helically twisted nanowires (Yang and Zhao, 2005). The framework thickness of the mesoporous silica is usually in the range of 2–11 nm; thus, the pore size of derived replicas of *m*-TMO is also in the same range (Schüth, 2001).

Ideally, all crystalline mesoporous materials can be synthesized using the hard-templating method if the corresponding metal precursors can be filled into the pore of the mold. So far, among numerous hard templates, mesoporous silica (e.g., SBA-15 and KIT-6) are commonly used for the preparation of mesoporous materials due to their diverse pore architectures and extremely uniform pore size/size distribution. SBA-15 has uniform hexagonal pores with the pore diameter in the range of 5–15 nm (Zhao et al., 1998a,b; Sakamoto et al., 2000). KIT-6 shows a bicontinuous structure as a gyroid cubic symmetry Ia3d with a pore diameter in the range of 4–12 nm (Kleitz et al., 2003). When using mesoporous silica as a template, the removal of silica to release the pore usually relies on the etching by concentrated NaOH or HF. One can also consider the use of mesoporous carbon as a template to prepare *m*-TMO, where the carbon template can simply be oxidized by low-temperature annealing in air (Lu et al., 2002; Roggenbuck and Tiemann, 2005; Polarz et al., 2007; Liang et al., 2008).

**Figure 2:**
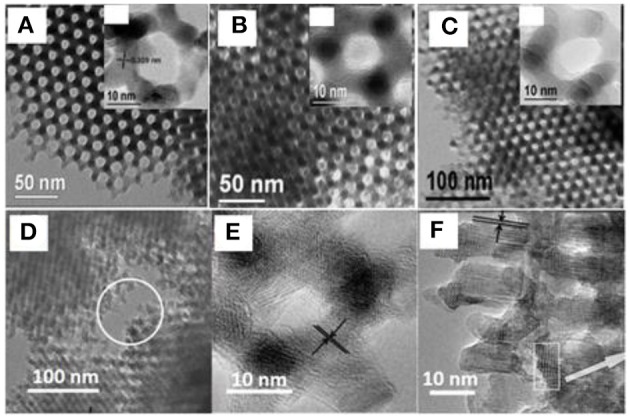
Control of crystallinity and mesostructures of Mn oxides. TEM images of **(A)** mesoporous β-MnO_2_, **(B)** Mn_2_O_3_, and **(C)** Mn_3_O_4_, and TEM images of **(D–F)** hierarchical porous MnO_2_ templated by KIT-6. Reprinted with permission from Jiao and Bruce (2007), Jiao et al. (2007) and Ren et al. (2013) Copyright 2013 Nature Publishing Group and Copyright 2007 Willey VCH.

Given the hard templates that can support and confine the grain growth of TMOs, the crystalline phases and pore features of metal oxides can be rationally designed. Taking manganese oxides as an example, Bruce's group synthesized mesoporous β-MnO_2_, Mn_2_O_3_, and Mn_3_O_4_ by high-temperature calcination at different temperatures or in the assistance of H_2_, using KIT-6 as the hard template (Jiao and Bruce, 2007; Jiao et al., 2007). All the materials possessed similar mesoporous frameworks and well-controlled crystalline phases of Mn oxides (Figures 2A–C). Similarly, iron oxides (Jiao et al., 2006) and cobalt oxides (Jiao et al., 2005a) with different crystalline phases were also obtained using the same strategy (Ren et al., 2009). Furthermore, bicontinuous mesoporous nanochannels of KIT-6 can also be utilized to tailor the final porosity of the materials. By selectively filling in different sets of nanochannels, mesoporous oxides with different pore sizes and even pore hierarchies (macropore, mesopore, and micropore) were successfully synthesized (Figures 2D–F) (Ren et al., 2013). The hard-templating synthesis of mesoporous materials is also extendable to metal sulfides and phosphides (Fu et al., 2016; Jin et al., 2017).

**Figure 3:**
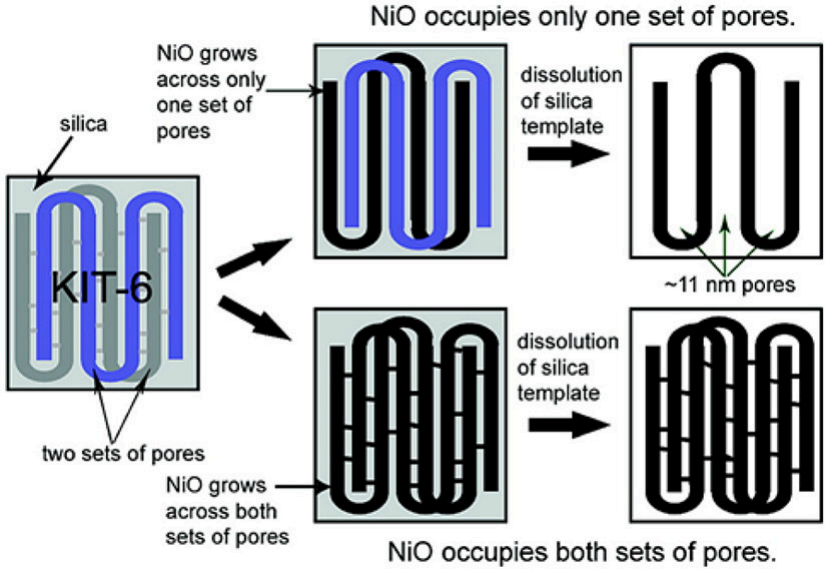
Scheme illustrating the synthesis of *m*-TMO with two pore size distributions by using KIT-6 as the template. Reprinted with permission from Jiao et al. (2008). Copyright 2008 American Chemical Society.

The hard-templating method shows limited control over the mesoporous structures but, in KIT-6 template, the distribution, and adjustable ratio of the two different pores can be achieved by hydrothermal synthetic conditions (Figure 3) (Jiao et al., 2008; Ren et al., 2010). Using mesoporous NiO as an example, an alcoholic solution of Ni(NO_3_)_2_ was mixed with KIT-6 and then redispersed in hexane to achieve a very high degree of pore filling. Followed by calcination at 550°C and removal of KIT-6 via etching with NaOH, mesoporous NiO with a specific surface area of 108.6 m^2^ g^−1^ can be synthesized. KIT-6 has two sets of pores with bicontinous channels and the development of these channels varies along with the hydrothermal synthetic conditions of KIT-6. At a higher hydrothermal temperature (>130°C), the existence of interconnected porous channels in KIT-6 favors complete growth of NiO across both sets of mesopores, leading to the formation of mesoporous NiO with a pore diameter (~3.3 nm) similar to the wall thickness of KIT-6 (Figure 3). On the other hand, with less interconnected pores at a lower hydrothermal temperature, the growth of mesoporous NiO within only one set of mesopores was observed. The resultant mesoporous NiO has a pore size of ~11 nm, equivalent to the dimension of two walls plus the pore size of KIT-6. Through this method, mesoporous β-MnO_2_ and NiO with large (~11 nm) and small (~3.3 nm) controllable pore sizes can be prepared.

Using SBA-15 as a template, *m*-TMO with ordered cylindrical channels can be prepared. Zhu et al. reported the highly crystalline Cr_2_O_3_ using aminopropyl-triethoxysilane (APTS)-modified SBA-15 (Zhu et al., 2003). The formation of crystalline Cr_2_O_3_ needs calcination of the Cr_2_O_3_@SBA above 350°C. After the removal of SBA-15, the mesoporous Cr_2_O_3_ possesses a specific surface area of 58.1 m^2^ g^−1^ and an average pore size of 3.4 nm. Other mesoporous *m*-TMOs with cylindrical pores can be prepared through a similar route (Chen et al., 2003; Jiao et al., 2005b; Yue et al., 2005). Our group also demonstrated mesoporous Co_3_O_4_, NiO, and their binary oxides by using bicontinuous KIT-6 as the template. Through a solid-phase sulfurization method based on sulfur, bicontinuous mesoporous CoS_2_, NiS_2_, and their binary sulfides can be further synthesized (Jin et al., 2017). When using the double templates, more sophisticated nanostructures can be designed. For example, if coated with thin carbon on the SBA channels first, the further growth of metal oxides will be confined in the hollow carbon nanotubes within SBA-15. Zhao et al. showed the synthesis of a mesoporous Co_3_O_4_@ carbon nanotube using double templates of silica and carbon. The resultant oxide-in-tube nanostructures possess a specific surface area up to 750 m^2^ g^−1^ and a controllable size of Co_3_O_4_ between 3 and 7 nm.

The synthetic challenge of the hard-templating method is to completely fill the pores of the template due to the slow mass transfer at nanoscale (Yang and Zhao, 2005). Mostly, only small pieces of mesoporous replicas can be yielded. Despite functionalized mesoporous templates with binding groups (e.g., -NH_2_, -OH, -CH = CH_2_) (Zhu et al., 2003; Tian et al., 2004; Wang et al., 2005), metal precursors still inevitably nucleate and grow in the outside of template pores, in particular under high temperature. Additionally, the pore size of resultant replicas that is determined by the thickness of the framework is usually small and less adjustable in both SBA series and KIT-6. Meanwhile, the nanocasting method is high-cost and time-consuming (Lu and Schüth, 2005, 2006; Yang and Zhao, 2005).

## Soft-Templating Method

The soft-templating method usually uses unrigid nanostructures formed by intermolecular interaction force as templates. Followed by the deposition of inorganic sources on the surface and interior of the unrigid soft templates, the formation of mesostructures with defined pore structures and sizes can occur. Generally, soft templates include soft matter, including organic surfactants and/or block copolymers, that can interact with metal ions and self-assemble into liquid crystal phases to template the sol-gel process. The assembled process between metal ions and soft matter is driven by weak non-covalent bonds, such as hydrogen bonds, van der Waals forces, and electrostatic interaction. In addition, cooperative assembly between metal ions and soft matter is mostly involved in facilitating this process. Mesostructures with open pores can be obtained after the removal of soft template through calcination. The soft-templating method offers a large room of controllability in terms of the pore structures and pore sizes compared to the hard-templating method. The key of the soft-templating method is the control over the sol-gel transition of precursors along the self-assembly of soft templates, i.e., surfactants/block copolymers (Meng et al., 2005; Li et al., 2013; Lokupitiya et al., 2016). By rationally tuning the cooperative assembly between precursors and templates, mesoporous materials can be obtained through either a solution-phase synthesis or evaporation-induced self-assembly (EISA) process. The first report on solution-phase synthesis of mesoporous oxides and phosphates by a similar route for MCM silica was in Huo et al. (1994). Due to the straightforwardness of tunability in size and self-assembled mesophases of surfactants/block copolymer, the soft-templating method has been widely used to tailor the mesoporous sizes and framework nanostructures of *m*-TMOs.

However, it is difficult to release mesopores by removing the surfactant because of the disruption and collapse of these porous frameworks during the crystallization of *m*-TMOs. Ying et al. first reported the ordered mesoporous TiO_2_ with open pores using titanium acetylacetonate tri-isopropoxide as Ti precursor and tetradecylphosphate as the template (Antonelli and Ying, 1995). The presence of acetylacetone slowed down the sol-gel transition, which allowed further interaction and co-assembly with Ti and the template. The resultant mesoporous TiO_2_ had a surface area of 200 m^2^ g^−1^ after calcination. Her group has extended this solution synthetic method to synthesize the ordered mesoporous Nb_2_O_5_ (Antonelli et al., 1996), VO_x_ (Liu et al., 1997), and ZrO_2_ (Wong et al., 1997; Antonelli, 1999).

Yang and Zhao creatively developed Pluronic polymers (commercial PEO-PPO-PEO triblock copolymers) as soft templates in non-aqueous solution to synthesize a series of mesoporous silica and metal oxide, including TiO_2_, ZrO_2_, Al_2_O_3_, Nb_2_O_5_, Ta_2_O_5_, WO_3_, HfO_2_, SnO_2_, and mixed oxides SiAlO_3.5_, SiTiO_4_, ZrTiO_4_, Al_2_TiO_5_, and ZrW_2_O_8_ (Yang et al., 1998, 1999). Pluronic polymers have a long flexible poly(ethylene oxide) (PEO) chain that can coordinate with transitional metal ions via weak coordination bonds. The absence of water enabled a controllable sol-gel, i.e., hydrolysis and condensation of metal precursors. These resulted in the optimal co-assembly at inorganic/organic interfaces; therefore, Pluronic polymers further templated the growth of a number of *m*-TMOs with periodically ordered mesoporous nanostructures. Through EISA, Sanchez et al. used PEO-based surfactants (e.g., Pluronic F127, Pluronic P123, Brij 56, and Brij 58) as templates to synthesize highly ordered *m*-TMOs (Grosso et al., 2001; Crepaldi et al., 2003). However, since the conventional EISA method usually employed Pluronic polymers as templates, the pore size of *m*-TMOs is usually <15 nm owing to the short hydrophobic PPO segments (Yang et al., 1998). Furthermore, both PEO and PPO polymers are thermally unstable. When annealing those *m*-TMOs to remove the template, the mesoporous nanostructures ineluctably undergo structural reconstruction for crystallization, leading to the overgrowth of crystal grains, and even collapse of mesoporous frameworks (Schüth, 2001; Yun et al., 2001).

**Figure 4:**
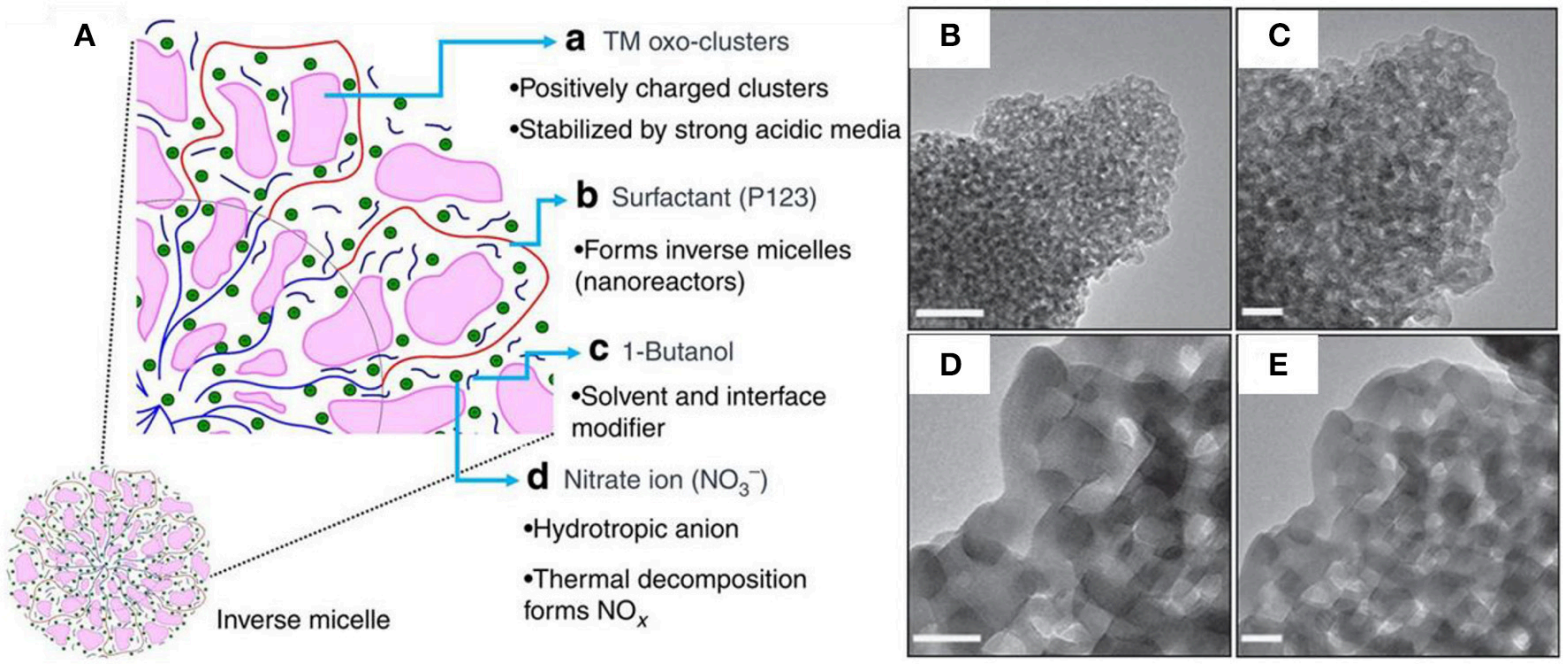
**(A)** The synthetic mechanism scheme of *m*-TMOs using the inverse sol-gel method. **(B–E)** HR-TEM images of mesoporous MnO_2_ at different calcination temperature: **(B,C)** 150°C and **(D,E)** 550°C. Scale bars are 20 nm. Reprinted with permission from Poyraz et al. (2013) Copyright 2013 Nature Publishing Group.

More recently, Suib's group has developed a universal strategy to synthesize the thermally stable and crystalline *m*-TMOs (Poyraz et al., 2013). As illustrated in Figure 4A, the inverse micelle formed by Pluronic P123 served as a nanoreactor where the positively charged transition metal-oxo clusters were confined via hydrogen bonding interaction. As stabilized by P123, the uncontrolled aggregation of the clusters was reduced and hindered. The addition of 1-butanol, the solvent and interface modifier, further enhanced the physical barrier between the oxo-clusters and prevented condensation. The essential presence of nitric acid in this system enabled the penetration of the positively charged metal-oxo clusters into the micelles, simultaneously increasing the solubility of P123 and hydrating the core. Interestingly, the decomposition of the nitrate ions into nitric oxides (NO_x_) played a critical role in the consecutive heat treatment by adsorption onto metal oxo-clusters, therefore impeding the uncontrolled condensation. After the removal of P123 by calcination at different temperatures ranging from 150 to 550°, the *m*-TMOs were readily obtained with tunable pore size distribution. For example, the mesoporous MnO_2_ evolved from amorphous materials with higher specific surface area (255 m^2^ g^−1^) and smaller pore size (2 nm) at a low temperature of 150° (Figures 4B,C), to the ordered crystalline Mn_2_O_3_ with lower specific surface area (35 m^2^ g^−1^), and enlarged pore size (12.3 nm) at an elevated temperature of 550° (Figures 4D,E). This method allows the general syntheses to a range of *m*-TMOs including nickel, cobalt, iron oxides etc (Poyraz et al., 2013, 2014; Song et al., 2014; Jiang et al., 2015; Wasalathanthri et al., 2015). It is worth mentioning that the series of the *m*-TMOs are highly resistant to elevated temperatures up to 400~550°, showing superior thermal stability. This unique stable mesoporous structure affords the development of more efficient and heat-tolerant catalysts in high-temperature reactions (Poyraz et al., 2013; Lu et al., 2015; Vovchok et al., 2018).

**Figure 5:**
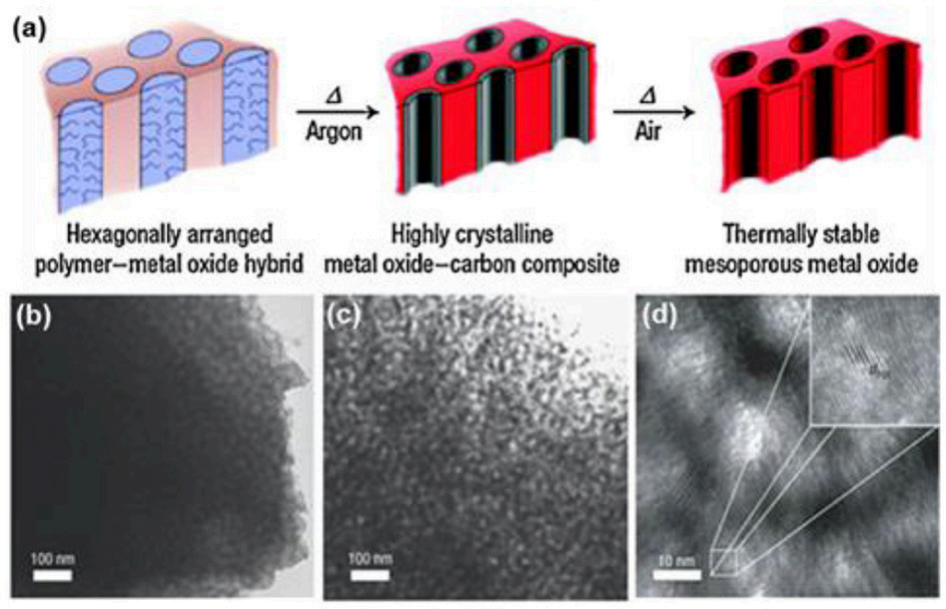
**(a)** Schematic illustration of combined assembly by soft and hard (CASH) method. TEM images of **(b)** as-made PI-*b*-PEO/TiO_2_ hybrids, **(c)** the TiO_2_-C composite after heat treatment under argon, and **(d)** the resultant *m*-TiO_2_ after removal of the carbon after calcination in air. Reprinted with permission from Lee et al. (2008). Copyright 2008 Nature Publishing Group.

As mentioned above, the crystallinity of TiO_2_ determines the surface amorphous defects that plays a critical role in photocatalysis. The high temperature treatment for a typical *m*-TiO_2_ obtained using Pluronic polymers as templates, usually causes structural collapse of the ordered mesoporous frameworks (Schüth, 2001; Yun et al., 2001). To solve the thermal stability challenges in soft-templating, Wiesner's group developed the combined assembly by soft and hard chemistries (so-called CASH, Figure 5a) method to fabricate mesoporous metal oxides (*m*-TiO_2_ and *m*-Nb_2_O_5_) with excellent thermal stability and crystallinity (Lee et al., 2008). In the CASH method for the synthesis of *m*-TiO_2_ (Figure 5a), titanium chloride firstly reacted with titanium isopropoxide to form the metal oxide clusters and corresponding alkyl halide, which was added into the tetrahydrofuran (THF) solution of the block copolymer of poly(isoprene-block-ethylene oxide) (PI-*b*-PEO, M_n_ = 27,220 g mol^−1^). The hydrophilic PEO selectively bound the metal oxide sol, which generated an amorphous hybrid material under evaporation of a PI-*b*-PEO/metal oxide solution in air (the obtained hybrid in Figure 5b). Secondly, the as-made PI-*b*-PEO/metal oxide hybrids were annealed at 700°C in argon to achieve the highly crystalline materials. The metal oxide crystals nucleate, grow, and sinter into porous frameworks and PEO was removed by thermal decomposition, while the PI segments can be transformed *in situ* into a carbon scaffold inside the pore channels due to the existence of *sp*^2^ hybridized carbon atoms in the PI segments of PI-*b*-PEO (the TiO_2_-C composite in Figure 5c). The generated carbon scaffold acts as a hard template *in situ* to prevent the collapse of mesoporous frameworks while crystallizing under high temperature treatment (700°C). Thus this generated highly crystalline TiO_2_ (Lee et al., 2008). Finally, the carbon inside TiO_2_-C composite could be removed by heating in air, yielding a well-organized, highly crystalline mesoporous TiO_2_ (Figure 5d) with a pore size of 23.6 nm and a specific surface area of 75 m^2^ g ^−1^. Other polymers, like poly(ethylene oxide)-*b*-polystyrene (PEO-*b*-PS), which includes *sp*^2^ hybridized carbon atoms and can be carbonized under inert atmosphere, potentially are useful to obtain highly crystalline *m*-TMOs using the CASH method (Zhang et al., 2011).

**Figure 6:**
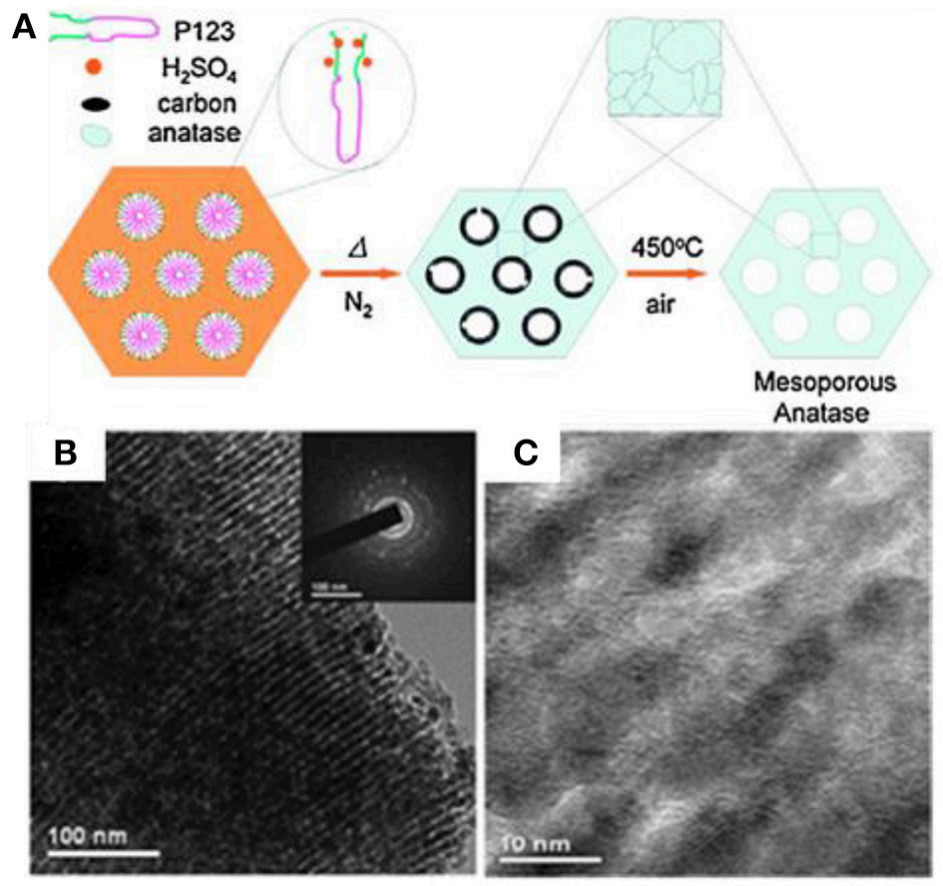
**(A)** Scheme showing the sulfuric acid carbonization method for the synthesis of *m*-TiO_2_. **(B,C)** TEM images of *m*-TiO_2_ prepared by the surfactant sulfuric acid carbonization method after calcinating at 650°C in N_2_. Reprinted with permission from Zhang et al. (2010). Copyright 20 Willey VCH.

Other than carbonization thermally, Zhao's group developed a simple sulfuric acid (H_2_SO_4_) carbonization method to generate a highly crystalline, ultra-stable ordered *m*-TiO_2_ by using Pluronic polymers (P123, F127, and F10) as templates (Figure 6A) (Zhang et al., 2010). In the process of EISA with titanium isopropoxide (TIPO), a mixture of HCl, and H_2_SO_4_ as the acidic catalyst carbonized the polymer template to form amorphous carbon in the original pore channels. The *in situ*-formed carbon templates can stabilize the mesoporous structures during crystallization (650°C) in N_2_. After burning off the carbon at 450°C in air, the resultant *m*-TiO_2_ with a specific surface area of 193 m^2^ g^−1^ and a pore size of 4.6 nm was generated, which also possessed a high crystallinity of anatase and thermal stability (Figures 6B,C). To keep the structural order of *m*-TMOs, Zhou et al. synthesized the well-ordered mesoporous *m*-TiO_2_ with improved crystallinity and remarkably thermal stability through the EISA method assisted with encircling ethylenediamine (EN) protectors to retain the mesoporous structure (Zhou et al., 2011). The EN species could tightly interact and bind on the surface of TiO_2_ while prohibiting undesirable grain growth and structure transformation during calcination. Thus, the mesoporous TiO_2_ obtained in this method is stable up to 700°C; and it has a specific surface area of 122 m^2^ g^−1^ and a pore size of 10 nm.

## Colloidal-Templating Method

The examples highlighted in the soft-templating method share a key idea, i.e., to improve the stability of hydrocarbon-based polymer templates and to confine the crystal growth during thermal treatment. Even though the carbonization of polymer templates enhances thermal stability, it is limited to some extent. The carbonized polymer templates require the thermal annealing with extra caution, for example, under inert gas atmosphere and without extremely long annealing time. Otherwise, the carbonized polymer templates can be oxidized, further resulting in the collapse of porous frameworks.

One ultimate solution to the thermal stability of templates is to introduce inorganic components, including inorganic nanoparticles (NPs) or non-hydrocarbon-based elements to polymer templates. Inorganic NPs have an inherently much higher thermal stability compared to organic polymers, while some of non-hydrocarbon-based elements, e.g., Si, can convert into inorganic oxides that are known to be thermally stable as well. By coupling inorganic templates with polymers, the advantages from soft-templating and hard-templating methods can be said to emerge as the “colloidal-templating” method. Colloidal templates are composed of an inorganic NP core tethered with flexible polymer tails. In view of the topology of colloidal templates, they are extremely like polymer micelles, which have been used in the soft-templating method to grow mesoporous materials (Bastakoti et al., 2014, 2016; Jiang et al., 2017, 2018; Tanaka et al., 2018). In the colloidal-templating method, polymers as soft templates are responsible for coordinating inorganic metal ions to self-assemble into nanostructures, similar to that of the soft-templating method. The inorganic NPs, on the other hand, serve as hard templates to offer thermal stability and nanoconfinement to crystallize the framework under high-temperature annealing. Colloidal templates similar to the surfactants/block copolymers can form periodically ordered nanostructures in the course of sol-gel to fabricate mesoporous materials.

One example of colloidal templates by Dong et al. is to synthesize mesoporous graphene using oleic acid (OA)-capped Fe_3_O_4_ nanocrystals (Jiao et al., 2015). The self-assembled OA-capped Fe_3_O_4_ first formed the ordered 3D Fe_3_O_4_ nanocrystal superlattices. The superlattices were further calcined at 1,000°C under argon. As inorganic components, Fe_3_O_4_ nanocrystals are thermally very stable with respect to annealing, while OA can be carbonized to form continuous carbon frameworks. Followed by the removal of Fe_3_O_4_ nanocrystals using HCl, the ordered mesoporous carbon was derived and then transformed into highly ordered mesoporous graphene with *fcc* symmetric mesoporosity. However, such a colloidal template is difficult to extend to the synthesis of *m*-TMO, since mixed metal oxides can be formed under high-temperature annealing.

**Figure 7:**
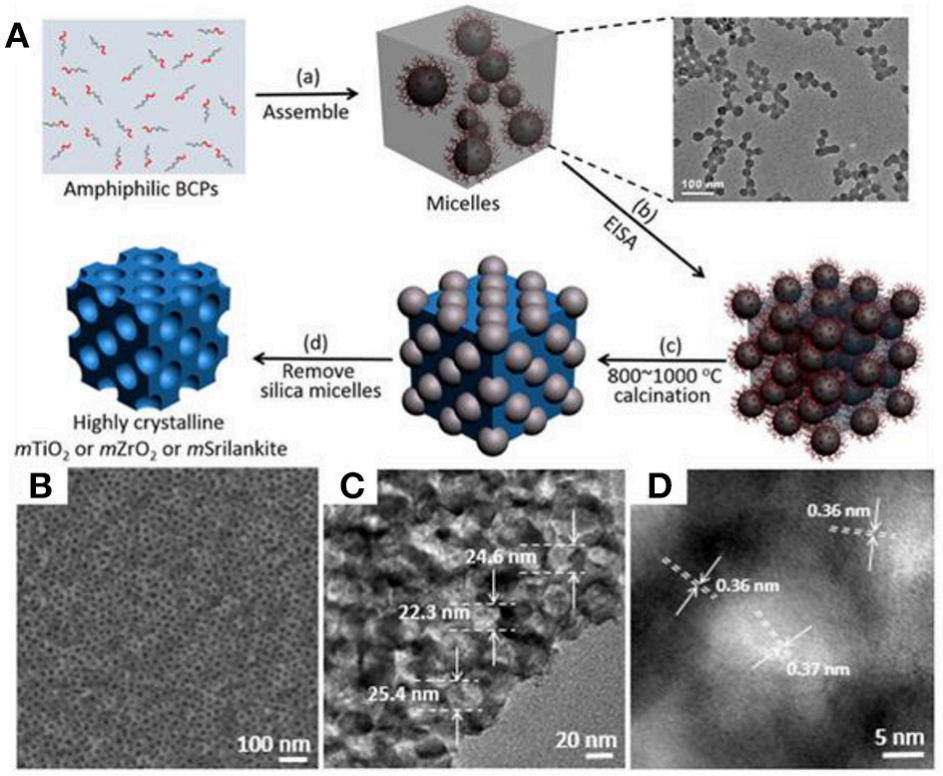
**(A)** Schematic illustration of highly crystalline *m*-TMOs based on the colloidal amphiphile-templating method. **(B)** SEM, and **(C,D)** TEM images of as-resultant *m*-TiO_2_ with uniform mesopores. Reprinted with permission from Liu et al. (2015b). Copyright 2015 American Chemical Society.

Organosilicate-containing colloidal templates are also utilized as soft-hard templates to synthesize highly crystalline mesoporous materials, which is largely beyond the capacity of the traditional soft-templating method. It is worth noting that the phase transition of TiO_2_ requires high temperature calcination (>800°C). Traditional carbon-based soft templates are unlikely to be stable at such a high temperature to support the mesoporous frameworks. Our group recently developed a new series of colloidal templates consisting of a silica-containing inorganic core and PEO shell (Liu et al., 2015b). The synthesis of such colloidal templates is based on the self-assembly of amphiphilic block copolymers in water (Figure 7). Briefly, a diblock copolymer of poly(ethylene oxide)-*block*-poly[3-(trimethoxysilyl)propyl methacrylate] (PEO-*b*-PTMSPMA) can form polymer micelles in the mixture solvent of water and ethanol. The micelles are highly uniform with an average diameter of 25 ± 2 nm. The hydrophobic PTMSPMA block contains trimethoxysilyl moieties that can hydrolyze to form polysilsesquioxane. Upon the addition of transition metal precursors, the coordination interaction between PEO and metal ions, similar to that in the soft-templating method, can result in the co-assembly of colloidal templates with metal precursors along with sol-gel transition during EISA. When annealing at high temperature, the polysilsesquioxane cores of colloidal templates further convert into rigid silica nanoparticles to support the structural integrity of the mesoporous framework, while the PEO will be burned off. Since silica is stable up to an air temperature of 1,000°C, the porous materials can be directly annealed at 1,000°C without disrupting their mesostructures. Therefore, the colloidal-templating method offers an ideal solution to the disadvantages of traditional soft templates, having poor thermal stability, and low mechanical strength.

Using *m*-TiO_2_ as an example, our group demonstrated that *m*-TiO_2_ synthesized with colloidal templates could be recrystallized at 900°C under air without further pretreatment (Figure 7B). *m*-TiO_2_ has a pore size of 21 nm and a specific surface area of 54.5 m^2^ g^−1^. Importantly, calcination at 900°C can lead to the formation of TiO_2_ with mixed anatase and rutile, similar to that of P25. Crystallization of mesoporous TiO_2_ is rationally tailored by calcination temperature or time, which exhibit dramatically enhanced activity toward photocatalysis (discussed below). In addition, the colloidal-templating method can be extended to synthesize the other *m*-TMOs, such as group-V *m*-ZrO_2_ and group-IV/group-V mixed transition-metal oxide, Zr_0.33_Ti_0.67_O_2_ (*m-*Srilankite). In particular, highly crystalline *m*-ZrO_2_ and *m-*Srilankite with uniform pore sizes can be obtained at 800°C.

Following the similar synthetic strategy, the colloidal templating method can used to prepare mesoporous carbon or TMO supported on carbon. To do so, the colloidal templates can template the oxidative self-polymerization of dopamine to polydopamine (PDA) nanospheres Liu et al. (2017b). In this case, the hydrogen bonds between dopamine with the PEO shell of colloidal templates drive the co-assembly. Since there are abundant functional groups (e.g., catechol and amine) of PDA to strongly coordinate metal ions (e.g., Co^2+^), the hybrids of PDA, and colloidal templates can physically absorb Co^2+^ ions. When annealing at 650°C under N_2_, the PDA backbone could be transformed into hierarchical porous carbon frameworks by pyrolysis while Co^2+^ ions were reduced metallic Co nanoparticles (2–7 nm) (Liu et al., 2017b). Here, the mechanically strong colloidal templates not only supported the porous carbon frameworks, but also confined the growth of Co NPs to restrain their overgrowth. Our group also extended this universal colloidal-templating method to develop a series of mesoporous hybrid nanostructures of metal-oxides and metal-carbons (Liu et al., 2016, 2017a,b, 2018).

**Figure 8:**
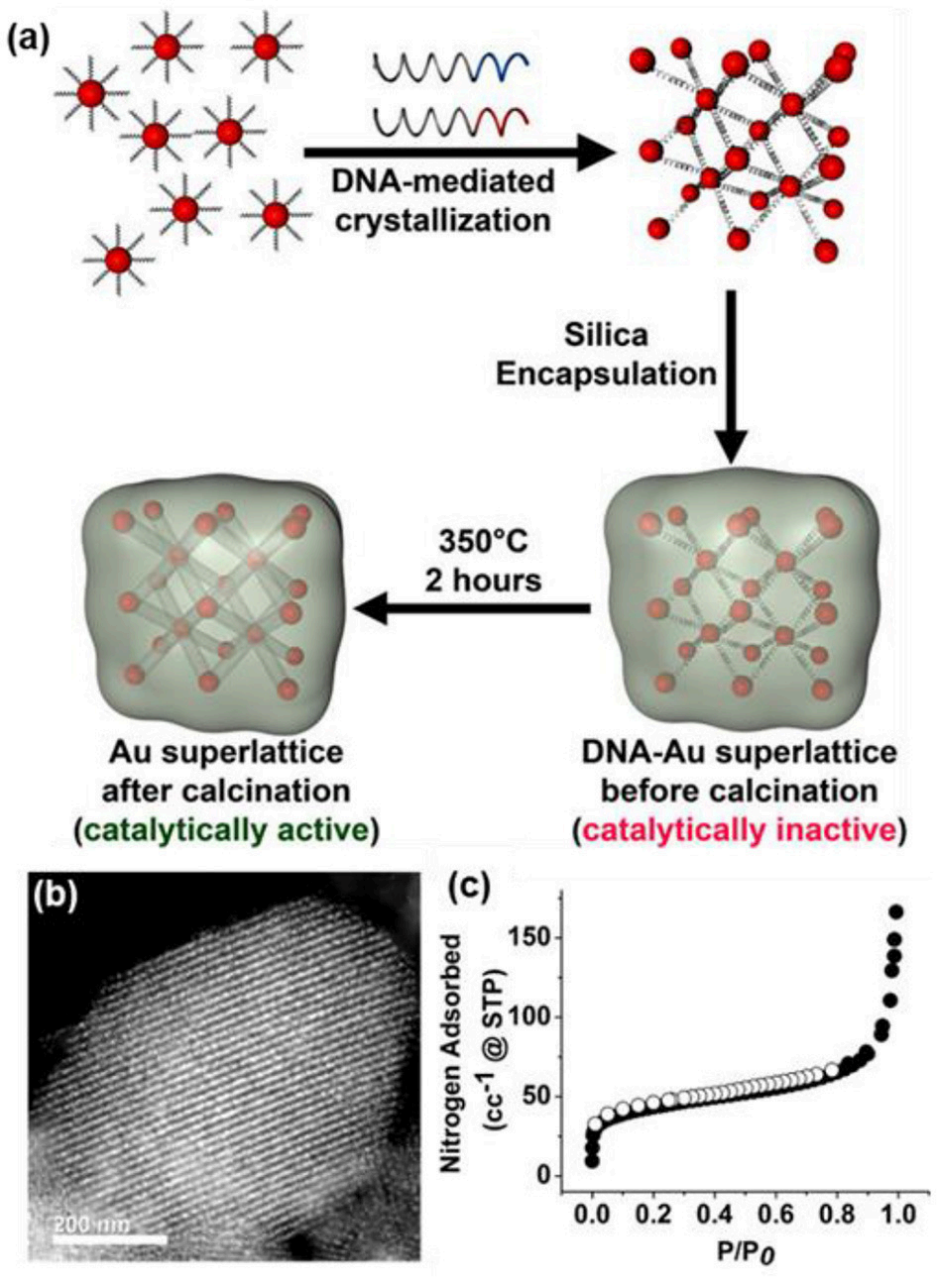
**(a)** Synthesis of the catalytically active DNA-NP superlattices. **(b)** The TEM image of silica-encapsulated Au superlattices after the removal of DNA. **(c)** Nitrogen sorption isotherms of porous silica. Reprinted with permission from Auyeung et al. (2015). Copyright 2015 American Chemical Society.

In addition, the use of colloidal templates can bring unexpected functionalities of inorganic NP to oxides, e.g., optical and/or catalytic properties, when replacing the silica cores of the colloidal template with noble metal NPs (Joo et al., 2009). One intriguing report from Mirkin's group shows that DNA-modified Au NPs as colloidal templates can guide the synthesis of mesoporous silica (Figure 8a) (Auyeung et al., 2015). To do so, the citrate-capped 5 nm Au NPs were first modified by thiolated DNA sequences (~25 dsDNA per NP). Other DNA strands complementary to the DNA ligands on Au NPs were added into the mixture solution (1:1 ratio) of Au to initiate rapid aggregation. The disordered aggregates were subsequently slow-cooled from 60 to 25°C at a rate of 0.1°C/10 min to get spatially organized Au superlattices. The Au superlattices were further utilized as templates to grow silica in the DNA domain. Upon the removal of DNA by calcination, Au NP superlattices within porous silica supports could be synthesized. Due to the confinement of silica, Au NPs have high thermal stability without sintering. The obtained hybrids show a typical Type-IV isotherm with a specific surface area of 210 m^2^ g^−1^ (Figures 8b,c). The porous Au NP superlattices with available channels are catalytically active in alcohol oxidation.

Co-assembly of two sets of colloidal templates into mesoporous materials also provides a new strategy to encapsulate functional NPs, especially noble metal NPs, within mesoporous frameworks of TMOs. Recently, two sets of colloids, including PS-*b*-PEO-tethered Au (Au-PS-PEO) and spherical colloidal of self-assembled PS-*b*-PEO, were utilized as co-templates for the growth of Au/*m*WO_3_ hybrid materials (Liu et al., 2015a). The two sets of colloidal templates share similar structural and chemical features and can co-assemble during the growth and crystallization of WO_3_, thus resulting in the homogeneous dispersion of Au NPs within *m*WO_3_. The porous frameworks can limit the mobility of Au NPs to enhance thermal stability, while the Au NPs confined by *m-*TMOs essentially exert plasmonic enhancement for the photocatalytic performance of *m-*TMOs. More importantly, because Au NPs are pre-synthesized, this method thus paves the first way to rationally control loading sizes and amounts of noble metal NPs within mesoporous oxides. The same strategy is also extendable to encapsulate noble metal NPs within *m*TiO_2_m (Liu et al., 2017a). Furthermore, two sets of the colloidal templates with different structural features are also successful in the formation of noble metal-oxide hybrid materials. For example, Au-PEO colloids and P123 have been used as co-templates to disperse Au NPs with mesoporous silica, which exhibited dramatically enhanced thermal stability of Au NPs for the high-temperature reaction (discussed later).

## Applications

**Figure 9:**
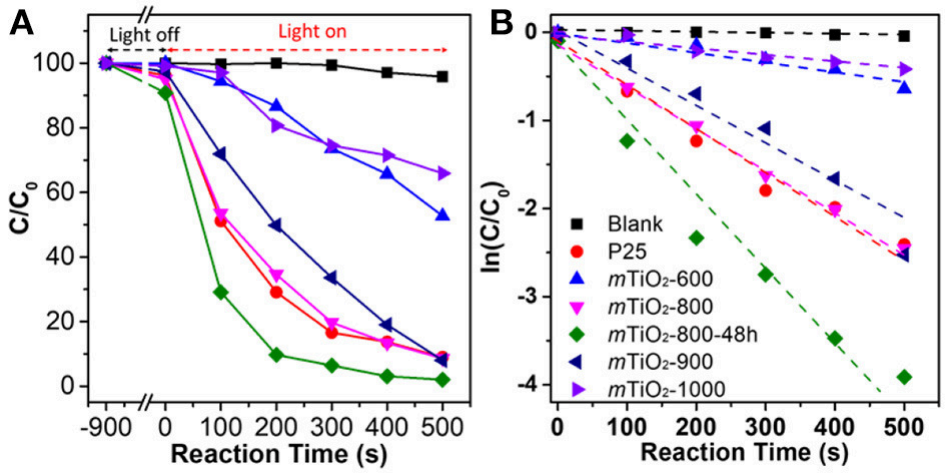
Photocatalytic activity of *m*-TiO_2_. **(A)** Photocatalytic degradation of RhB and **(B)** the fitting of the rate constant by using different mesoporous TiO_2_ (*m*TiO_2_) obtained at different calcination temperatures. Reprinted with permission from Liu et al. (2015b) Copyright 2015 American Chemical Society.

Crystalline *m*-TMOs with high surface areas, uniform pore sizes, and accessible interspaces exhibited great potential in many applications. We highlight a few examples on the applications of *m*-TMOs synthesized via the colloidal-templating method. The first example of the application is *m*-TiO_2_ synthesized by organosilane-containing colloids for photocatalytic degradation of organic dye (Liu et al., 2015b). As discussed above, having thermally stable colloidals as the soft-hard templates renders the resultant *m*-TiO_2_ with the precisely tunable crystalline phase of anatase and rutile by tuning the calcination temperature. The percentage of anatase and rutile can be controlled by calcination temperature and annealing time. The mesoporous TiO_2_ obtained at 1,000°C for 1 h has ~39% of rutile and ~61% of anatase. In contrast, the mesoporous TiO_2_ obtained below 800°C has almost 100% anatase phase. Both crystallinity and controllable anatase/rutile interface play an important role in the enhancement of photocatalytic activity. We used the photo decomposition of organic dye (Rhodamine B, RhB) to evaluate the photocatalytic performance of different *m*-TiO_2_ obtained from different calcination conditions. The photocatalytic decomposition results of RhB were obtained from the change of absorption intensity in the corresponding UV-vis spectra (Figure 9A). In addition, Figure 9B displays the kinetic fitting results by plotting ln (C/C_0_) against the reaction time. The control experiment without a catalyst showed a very slow decomposition rate of RhB, with a rate constant of 9 × 10^−5^ s^−1^. *m*-TiO_2_ obtained at 800 and 900°C exhibited an increased photocatalytic activity with rate constant of 5.1 × 10^−3^ s^−1^ and 4.4 × 10^−3^ s^−1^, respectively. The photo-decomposition activity of *m*-TiO_2_ obtained at 800°C for 48 h with the highest activity showed 1.7 times higher than that of commercial P25. The above results indicate that the importance of mesoporous nanostructures and the interface of anatase and rutile phases for their photocatalytic activity. The change of photocatalytic activity is closely related to the crystallinity and controllable interface of anatase/rutile. The highly crystalline *m*-TMOs also can be prepared by using the CASH method (Lee et al., 2008), which decreased the defects to improve performance in photocatalysis.

**Figure 10:**
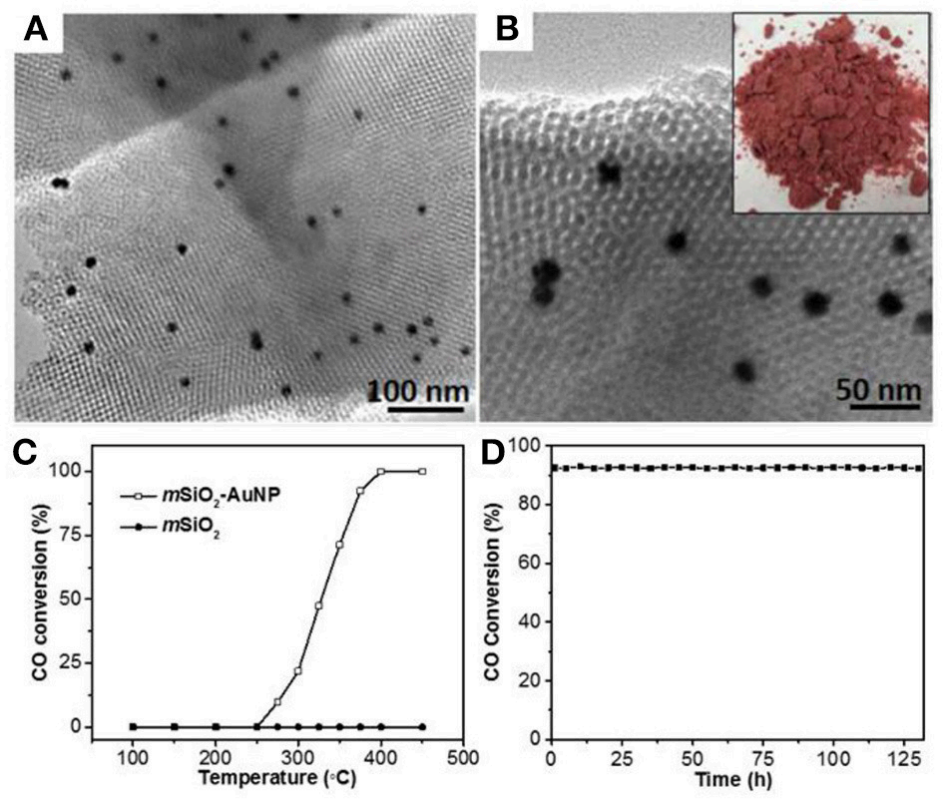
**(A,B)** TEM images of Au@*m*SiO_2_. The size of Au NPs is 14 nm. **(C)** High-temperature CO oxidation performance of mesoporous SiO_2_ (*m*SiO_2_) and mesoporous SiO_2_ with 1% loading of 14 nm Au NPs (*m*SiO_2_-AuNP14-1%). **(D)** Durability test of CO oxidation with *m*SiO_2_-AuNP14-1% at 375°C over 130 h. Reprinted with permission from Liu et al. (2017a). Copyright 2010 Royal Society of Chemistry.

The colloidal-templating method provides a new avenue to stabilize the noble metal NPs within a mesoporous framework, especially with amorphous SiO_2_ as the support. Using KIT-6-typed SiO_2_ with the framework and mesochannels smaller than the size of Au NPs could dramatically enhance the thermal stability of Au under high temperature, while Au NPs confined by mesoporous frameworks remain accessible and catalytically active (Liu et al., 2017a). We evaluated the high temperature catalytic performance of Au@*m*SiO_2_ (Figures 10A,B) for CO oxidation. As shown in Figure 10C, when the catalytic temperature reached 275°C (light-off temperature), the CO conversion of *m*SiO_2_-AuNP hybrids show catalytic activity for CO oxidation and achieved 100% of CO conversion at 400°C. In contrast, *m*SiO_2_ without Au NPs showed no CO oxidation activity even at 450°C. The high temperature stability of *m*SiO_2_-AuNP hybrids is further investigated in Figure 10D. No decrease in activity was observed for more than 130 h of continuous operation, indicating that *m*SiO_2_-AuNP hybrid catalysts have outstanding stability under high temperature. Not only the mesoporous structure of Au@*m*SiO_2_, but also Au NPs displayed extraordinary stability after CO oxidation for 130 h, further indicating the superior thermal stability of Au endowed by the nanoconfinement effect of mesoporous oxide supports. Moreover, these mesoporous metal-oxide hybrids are also potentially useful for other high temperature catalysis such as the oxidation of methane to methanol (Agarwal et al., 2017; Williams et al., 2018).

## Summary and Perspectives

Advances in the rational design and formation of highly crystalline *m*-TMOs with controlled crystallinity and functions largely broaden their practical applications in catalysis and energy storage/conversion. The development of new synthetic methods is the key for all those applications. In this review, we summarize the current synthetic methodologies of *m*-TMOs, including soft-templating, hard-templating, and colloidal-templating methods, and briefly discuss their advantages/disadvantages for the synthesis of *m*-TMOs. Special focus is given to the colloidal-templating synthesis of *m*-TMOs and hybrid materials of porous metal-oxides, beyond the capability of other traditional synthetic methods. Several typical examples are provided to highlight the potential applications of *m*-TMOs and porous metal-oxides synthesized by colloidal-templating method.

Despite much progress made in the synthesis of crystalline *m*-TMOs, there are still unmet synthetic challenges, particularly for the colloidal-templating method. First, no example has been documented on crystalline perovskites using soft-templating or colloidal-templating methods. Perovskites, as a complex oxide consisting of two or more simple oxides, have a cubic structure with a general formula of ABO_3_; and they have been studied extensively due to the interesting electronic and catalytic activity, e.g., as a solid electrolyte (Zhou et al., 2016) and cathode materials for fuel cells (Skinner, 2001; Suntivich et al., 2011). The synthesis of perovskites usually relies on the solid-state annealing of the mixture of oxides at >700°C (Zhu et al., 2014). Because of the slow diffusion in solid states, the synthesis of perovskites can take a few hours to days to anneal the oxide mixtures. With the new CASH or organosilicate-containing colloidal-templating methods, it will be of interest to extend the current synthetic capacity to crystalline perovskites. Secondly, encapsulating noble metal NPs within highly crystalline *m*-TMOs is also unresolved. There is an extensive literature on the strong metal-oxide interaction (Tauster, 1987; Farmer and Campbell, 2010), known to be critical for catalytic activity of metal-oxide hybrids. The formation of the strong metal-oxide interaction (or strong metal-support interaction, SMSI) requires the annealing under reductive atmosphere. Control of metal-oxide interaction in the context of mesoporous hybrids has rarely been reported in previous research. In addition, we expect that more achievements from other fields, including macromolecules, electronics, and biomedical, can be incorporated in further developments of the synthesis and applications of *m*-TMOs. For example, new colloidal templates with different topologies assembled from different amphiphilic block copolymers (Yao et al., 2015) can add more structural hierarchies to crystalline *m*-TMOs. The interdisciplinary aspects that bring the new knowledge across fields will create new opportunities in solving those synthetic challenges.

## Author Contributions

All authors listed have made a substantial, direct and intellectual contribution to the work, and approved it for publication.

### Conflict of Interest Statement

The handling editor declared a shared affiliation, though no other collaboration, with the authors JH, LJ, and LZ at the time of review. The remaining author declares that the research was conducted in the absence of any commercial or financial relationships that could be construed as a potential conflict of interest.
